# From the field: Educating DR patients on gaining better diabetes control

**Published:** 2015

**Authors:** Zhara Gregory

**Affiliations:** Diabetes Specialist Nurse: Homerton, Diabetes Centre, London, UK. zhara.gregory@nhs.net

As part of the Vision 2020 Links Programme, a team from the Diabetes Eye Screening Department at Homerton University Hospital in London, UK visited the University Hospital of West Indies (UHWI) in Kingston, Jamaica in June 2015. The purpose of the visit was to share the Homerton team's experiences with the UHWI's ophthalmic department in support of their plans to start a pilot eye screening programme for diabetic patients. If it works, this programme would be implemented across the whole island in five years' time.

Since I am experienced in retinal grading and work as a diabetes specialist nurse, my contribution to the team was to share clinical skills on the management of diabetes with the ophthalmic nurses who were going to set up and run the programme.

It is generally accepted that poor control of diabetes leads to complications such as diabetic retinopathy (DR). Tackling this requires a dual approach: eye screening programmes that can detect diabetic retinopathy early and ensure the disease is treated in its early stages before it can progress to more advanced retinopathy and blindness; and the input of diabetes teams in helping patients to achieve better control of their blood pressure, blood glucose and cholesterol levels. Evidence from the United Kingdom Prospective Diabetes Study (UKPDS)^1^ and Diabetes Control and Complications Trial (DCCT)^2^, suggest that controlling blood glucose and blood pressure is crucial in reducing the risk of developing sight-threatening DR. Other studies have suggested that hard exudates and macular oedema are often associated with high lipid levels.^3^

In my discussions with the ophthalmic nurses in Kingston, it emerged that patients receiving retinal grading results indicating early retinopathy were likely to face a long wait to be seen by the diabetes team. The ophthalmic nurses were keen to learn more about how to advise their patients about immediate lifestyle changes that patients could implement during their wait for more specialist input. I share some of this advice in the article ‘Information for patients: living with diabetes and protecting your eyes’.

Patients will often want to make changes to their lifestyle or gain better control of their diabetes once they know that they are starting to get the complications of the disease. If we work with patients who are undergoing retinal screening/grading, we are in a good position to discuss their results with them and also to be the first port of call to offer advice on what patients can do until they see their diabetes team.
